# Does Metabolically Healthy Obesity Exist?

**DOI:** 10.3390/nu8060320

**Published:** 2016-06-01

**Authors:** Araceli Muñoz-Garach, Isabel Cornejo-Pareja, Francisco J. Tinahones

**Affiliations:** 1 Department of Endocrinology and Nutrition, Virgen de la Victoria Hospital, Málaga University, Málaga 29010, Spain; isabelmaria_cornejo@hotmail.com (I.C.-P.); fjtinahones@hotmail.com (F.J.T.); 2 CIBER Fisiopatologia Obesidad y Nutricion (CIBEROBN), Instituto de Salud Carlos III, Madrid 28029, Spain

**Keywords:** metabolically healthy obesity, metabolic syndrome, adipose tissue, inflammation

## Abstract

The relationship between obesity and other metabolic diseases have been deeply studied. However, there are clinical inconsistencies, exceptions to the paradigm of “more fat means more metabolic disease”, and the subjects in this condition are referred to as metabolically healthy obese (MHO).They have long-standing obesity and morbid obesity but can be considered healthy despite their high degree of obesity. We describe the variable definitions of MHO, the underlying mechanisms that can explain the existence of this phenotype caused by greater adipose tissue inflammation or the different capacity for adipose tissue expansion and functionality apart from other unknown mechanisms. We analyze whether these subjects improve after an intervention (traditional lifestyle recommendations or bariatric surgery) or if they stay healthy as the years pass. MHO is common among the obese population and constitutes a unique subset of characteristics that reduce metabolic and cardiovascular risk factors despite the presence of excessive fat mass. The protective factors that grant a healthier profile to individuals with MHO are being elucidated.

## 1. Introduction

Obesity is a major health problem and an important risk factor for the development of diseases such as diabetes mellitus. The mechanism by which obesity leads to the development of insulin resistance still needs to be elucidated. There is a large body of epidemiological evidence on the relationship between obesity and other metabolic diseases. However, there are clinical inconsistencies that are exceptions to the paradigm of “more fat, more metabolic disease”. There are subjects with long-standing obesity and morbid obesity who can be considered healthy despite a high degree of obesity. This phenomenon was described 15 years ago [1], and these subjects were referred to as metabolically healthy obese (MHO).

There is great inconsistency in the definitions of MHO, with a high degree of variability surrounding the prevalence of this phenotype, which has been estimated to be between 10% and 34% depending on the criteria used [2,3,4,5]. MHO appears to be more prevalent in women than in men and its prevalence appears to decrease with age in both sexes [6]. At the same time, there are individuals who, despite having “normal” weight, have an increased risk of disease.

Historically, the primary concern regarding obesity was the concurrent metabolic and cardiovascular risk. In recent years, there has been increased awareness of those individuals who do not fit into this traditional phenotype. This suggests that fat storage is not the only determinant in the association between obesity and insulin resistance, and the term “adiposopathy” is beginning to be used.

Existing guidelines also fail to individualize the management of MHO or metabolically unhealthy/abnormal obese (MUO) patients. This is further complicated because of a gap in the recognition and appropriate management of those normal-weight individuals who demonstrate high metabolic risk profiles.

## 2. Definition of Metabolically Healthy Obesity

Most studies suggest the definition of MHO (body mass index (BMI) ≥ 30 kg/m^2^) to be obesity without the presence of metabolic diseases such as type 2 diabetes (T2DM), dyslipidemia or hypertension [1,7,8]. To date there are no accepted criteria for identifying MHO [7] individuals. The identification of individuals with MHO is hampered by the absence of a standardized definition of the condition. Several approaches were used to identify or define the MHO phenotype. The most referenced are:

1. Hyperinsulinemic-euglycemic clamp [8,9,10].
2. The upper quartile of glucose disposal rate [10].
3. The upper quartile of an index of insulin sensitivity after an oral glucose tolerance test [11,12].
4. Less than two of the following cardiometabolic disorders (systolic blood pressure ≥130 mmHg, diastolic blood pressure ≥85 mmHg, triglycerides ≥1.7 mmol/L or ≥150 mg/dL, fasting glucose ≥5.6 mmol/L, homeostasis model assessment of insulin resistance (HOMA-IR) ≥5, ultrasensitive C-reactive protein (CRP) ≥0.1 mg/L, HDL cholesterol ≤1.03 mmol/L or 40 mg/dL in men and ≤1.3 mmol/L or 50 mg/dL in women [3,13].
5. Less than three metabolic syndrome criteria [14].
6. Subjects with BMI above 30 kg/m^2^ and HDL cholesterol levels of at least 40 mg/dL in the absence of T2DM and hypertension [15].

Other inflammatory markers (CRP or degree of leukocytosis) have been suggested for inclusion in the definition of MHO [16,17].

An additional marker that is increasingly important in the context of the MHO phenotype is liver fat content. The prevalence of non-alcoholic fatty liver disease appears to be significantly lower in patients with MHO compared with MUO individuals [12]. According to our current knowledge, the classification of “metabolically benign obesity” or “metabolically healthy obesity” only refers to metabolic or cardiovascular complications and does not consider that obesity may be associated with other non-metabolic complications such as orthopedic problems, pulmonary complications, or other physiological conditions. Considering that the descriptions of MHO are inconsistent, the elucidation of the factors or mechanisms underlying this protective profile is far from complete although some works are trying to establisha standard definition of MHO [18].

## 3. Underlying Mechanisms That Explain the Existence of MHO

### 3.1. Subclinical Inflammation

Inflammation promotes insulin resistance. Greater adipose tissue inflammation is closely associated with increased metabolic risk for T2DM, cardiovascular disease, and fatty liver disease, whereas obese adults without adipose tissue inflammation exhibit reduced metabolic risk.

There is growing evidence to suggest that subclinical inflammation may be the underlying mechanism that determines whether or not an individual is MHO [16]. Subclinical inflammation is associated with insulin resistance, and CRP has emerged as one of the best predictors of vascular inflammation, metabolic syndrome and cardiovascular disease [19]. There is a strong correlation between circulating CRP levels and anthropometric markers and body composition, and CRP has been proposed as a screening tool to assess the risk of the metabolic syndrome in youth [20]. In adulthood, the MHO phenotype is associated with low levels of complement component 3, CRP, alpha necrosis tumor factor (TNF-α), interleukin 6 (IL-6), and a low number of white blood cells, supporting the concept of a more favorable inflammatory state compared to non-MHO subjects [21].

On the other hand, anti-inflammatory adipokines, such as adiponectin, IL-4, IL-10, IL-13, IL-1 receptor antagonist, and transforming growth factor beta are abundant within the adipose tissues of lean individuals, whereas obese adipose tissue is dominated by the release of pro-inflammatory adipokines, including leptin, resistin, TNF-a, IL-6, IL-18, retinol-binding protein 4, lipocalin 2, angiopoietin-like protein 2, CC-chemokine ligand 2, CXC-chemokine ligand 5, and nicotinamide phosphoribosyltransferase [22].

Other systemic inflammation markers or adipose tissue inflammation markers are free fatty acids (FFA) and the number and activation status of peripheral leukocytes. Thus obese patients with T2DM have higher circulating levels of IL-6, FFA and glycerol and a greater absolute number of peripheral leukocytes compared with non-diabetic obese subjects [23,24]. Similarly, our group found that morbidly obese subjects without metabolic disease had a low degree of insulin resistance and identical adipose tissue inflammation markers as those of non-obese subjects, with no infiltration of macrophages and elevated values of TNF-α and IL-6 expression in visceral adipose tissue [25]. All these results suggest that MUO patients show a higher degree of both systemic and local fat inflammation compared with MHO individuals [23].

### 3.2. Expansion Capacity of Adipose Tissue

Previously, to explain the transition from normal adipose tissue to that which leads to metabolic abnormalities, studies proposed the “adipose tissue expandability hypothesis”. This postulated that once adipocytes reached a threshold capacity for storage, they begin to promote insulin resistance with lipotoxicity and adipokine release. This was supported by knockout studies with PPARγ, lipodystrophy models, and alterations in adipokine secretion following saturation of adipose tissue. Investigating this fact led to further characterization of genetic contributors to the pathways of adipogenesis (SFRP1, Wnt, S14), apoptosis (TRAIL, TWEAK, BCL2, CASP3/7), and angiogenesis (VEGF-A, -B,-C,-D). To improve the understanding of adipose tissue in the context of MHO patients, Tinahones *et al.* looked at genes associated with both lipolysis and lipogenesis and BMI, insulin, and HOMA-IR. They found a positive correlation in PPARγ, DGAT1, AQP7, GK, ATGL, HSL, and perilipin and BMI, insulin, and HOMA-IR in both subcutaneous and visceral fat tissues. Additionally, they demonstrated a negative correlation between genes—ACC1, PEPCK, ACSS2, FABP4—and the aforementioned measures of metabolic risk.

A fairlysolid theory attributes the differences between MHO and MUHO subjects to the different capacity for adaptation to excess energy in adipose tissue [26]. When increased fat storage is required, fat tissue needs to increase its storage capacity and can increase the size of adipocytes or increase their number [27]. In addition, this increase in the overall amount of fat must be accompanied by increased vascularization [28]. In those subjects in whom adipose tissue has difficulty expanding in the healthiest way, which is increasing cellularity, metabolic disease does appear [29]. That is what we see in MHO who keep the proper storage capacity. But the loss of expansion capacity can even occur in patients with normal weight, so this theory would explain the existence of metabolically unhealthy lean subjects [30]. Furthermore, the lack of expansion capacity of adipose tissue has been linked to the loss of its main lipogenic function, and the appearance of lipotoxic products in adipose tissue [31].

Traditionally it was thought that there was little cell turnover in adipose tissue in adulthood. Today it is known that adipose tissue is capable of hyperplasia throughout life, and stem/progenitor cells are responsible for this adipose tissue hyperplasia [27,32,33]. Pre-adipocytes found in the vascular stroma are progenitor cells that differentiate into mature adipocytes through a complex program of gene expression [33,34,35,36]. Another variable that is directly related to the total number of fat cells is cell death, which may occur by necrosis, autophagy or apoptosis [37,38]. These studies have also alluded to reduced adipocyte hypertrophy, fibrosis and stress as potential contributors to this presentation. Therefore, there are four factors that are directly related to the ability for adipose tissue expansion and functionality: (1) lipogenesis; (2) adipogenesis via the newly formed progenitor cells; (3) apoptotic and anti-apoptotic pathways; and (4) angiogenesis.

Our group, in collaboration with other groups, has shown that metabolically healthy subjects have higher lipogenic [39,40] and angiogenic [41,42] capacity than metabolically unhealthy patients. Furthermore, there is also considerable evidence that adipogenesis and the functionality of mesenchymal cells to become new adipocytes is higher in metabolically healthy subjects because they exhibit mesenchymal cells in the stroma of adipose tissue with a greater capacity to differentiate both bone and fat tissue and a lower senescence [36]. This increased ability to form new adipocytes in the MHO individuals is an observation contributed by our group which clearly shows that the apoptosis of adipocytes increases with obesity, and this has a direct relationship with the degree of adipose tissue inflammation [43].

Adipose tissue is a key regulator of inflammation, and inflammation is involved in the onset and development of atherosclerosis, metabolic syndrome, and T2DM. Although the results of these initial studies suggest that MHO is characterized by a lower inflammatory cytokine environment than MUO, further studies are necessary to better identify the adipose tissue hormones that promote a healthy metabolic profile in obese adults.

There are several adipose tissue compartments and they have different connections with metabolic diseases. Visceral and intrahepatic fat has a direct association with obesity, while subcutaneous fat is not associated with metabolic disease [44]. A plausible hypothesis is that the healthiest way to accumulate fat is through subcutaneous fat expansion but when this capacity is decreased or lost it must resort to other compartments and metabolic diseases occur. Could the MHO phenotype be defined as a group of obese patients with predominantly subcutaneous fat deposits and with little or no visceral and intrahepatic fat? These questions and more still need to be answered.

### 3.3. Other Possible Mechanisms

A new potential factor to explain the differences between MHO and MUO could be circulating microRNA [45]. Insulin resistance in peripheral organs, such as the liver, can develop due to toxic stimulus, initiating secretion of microRNAs associated with insulin resistance, which are incorporated into muscle and fat cells, inducing insulin resistance in these tissues [46].

A study revealed that 20% of patients with a BMI above 40 kg/m^2^ had adiponectin levels above the average of subjects with normal BMI, and these adiponectin levels above a certain threshold increase the probability of being metabolically healthy, even after correcting for the confounding effects of age, insulin and waist circumference [15]. This suggests that adiponectin plays a crucial role in the pathogenesis of metabolic complications associated with obesity as patients with the MHO phenotype have similar levels of adiponectin as those found in normal-weight subjects [15]. Furthermore, individuals with the MHO phenotype have a lower visceral fat, liver fat, and muscle fat content than obese subjects without MHO or obese people with insulin resistance, suggesting that the MHO phenotype is associated with improved ability to capture FFA in adipose tissue [12]. These differences in body composition between MHO patients and non-MHO patients are consistent between genders [3].

Finally, lifestyle factors such as level of physical activity or cardiorespiratory condition also appear to play a key role in distinguishing whether or not an individual is MHO. MHO subjects show higher levels of physical activity compared to MUO individuals and have a more favourable lifestyle [14,47,48]. Likewise, occupational physical activity or leisure time physical activity also appears to be important, since the two exercise regimens are differentially associated with obesity and insulin resistance [49].

## 4. Do Metabolically Healthy Obese Subjects Improve after an Intervention?

The decisive feature of MHO is the absence of visceral fat accumulation. Until definitive data emerges linking genetic predisposition to MHO, currently recommended lifestyle modifications appear beneficial. Thus, promoting lifestyle modifications directed at minimizing visceral fat accumulation is a fundamental public health measure.

However, it is not completely clear if obese subjects with a MHO phenotype would benefit from traditional lifestyle interventions, which focus on dietary therapy and/or increased physical exercise. Few studies have analyzed the metabolic effects of lifestyle modifications with a restrictive diet and/or exercise in MHO subjects, and their results have been contradictory [50,51]. Supporting the theory that MHO and non-MHO individuals could require a different treatment approach, Karelisy *et al.* showed that MHO individuals reacted differently, from a metabolic viewpoint, to a six-month calorie restricted diet compared to “at-risk” obese people despite achieving similar weight loss [52]. There are some studies that showed combining a Mediterranean diet with moderate to high intensity aerobic training is more effective at improving body composition [53,54]. Although the public health message for all obese patients should continue to promote healthy lifestyle habits, the controversial results of lifestyle interventions in MHO individuals would justify prioritizing, for cost-efficacy reasons, the intensive interventions in metabolically abnormal obese individuals and monitoring MHO subjects for early detection of the development of metabolic abnormalities [55].

Currently, bariatric surgery remains among the best options for patients suffering from severe obesity, with excellent results regarding long-term weight loss and decreases in peripheral and visceral fat depot sizes [56]. More research is needed to study the effects of different therapeutic approaches on the metabolic profile of healthy but obese individuals. Results in this area are essential for understanding the pathophysiology and to implement appropriate metabolically healthy obese and healthy non-obese intervention strategies.

## 5. Do Metabolically Healthy Obese Subjects Stay Healthy as the Years Pass?

Debate continues concerning whether individuals with MHO are truly healthy. The literature differs regarding the relative risk of disease among this population. MHO individuals are at decreased risk for developing cardiovascular disease compared with MUO individuals [57]. Long-term studies have suggested that MHO is a transient state. For example, among a group of Japanese Americans with MHO, two-thirds developed metabolic syndrome during 10 years of observation, and the metabolic abnormality was independently associated with visceral fat accumulation, female sex, higher fasting plasma insulin concentration, and lower serum HDL associated cholesterol concentration [58]. Consistent with this report, among the 1051 participants in the Pizarra Study, the prevalence of MHO decreased during 11 years of observation [59].

Individuals with MHO are not at increased risk for developing cardiovascular disease compared to metabolically healthy normal weight [MHNW] individuals [60,61,62,63]. Studies with longer follow-up periods (>15 years) have reported that MHO individuals were at an increased risk for major cardiovascular disease events as compared to MHNW individuals [64,65]. Recently, analysts conducting a systematic review of the associations between BMI and metabolic status with total mortality and cardiovascular events reported that MHO individuals appear to be at increased risk for cardiovascular events, as compared to MHNW individuals [66]. These researchers concluded that obese individuals are at increased risk for adverse long-term outcomes, even in the absence of metabolic abnormalities, compared with MHNW individuals.

A recent study evaluated the prevalence of elevated plasma high sensitivity CRP (hs-CRP) concentrations and hepatic steatosis in MHO, MHNW, and in metabolically unhealthy normal-weight (MUNW) individuals [67]. They observed that both elevated plasma hs-CRP concentrations and hepatic steatosis are more prevalent among MHO and MUNW individuals than they are among MHNW individuals. However, they are most prevalent among individuals with MHO, suggesting that obesity in the absence of metabolic risk factors is not entirely benign but is associated with subclinical vascular inflammation. Another recent study reported that 42% of their subjects with MHO developed the metabolic syndrome within 10 years [68], again suggesting that MHO is not without increased health risks. However, the identification of predictors, biological determinants, and mechanisms underlying MHO, determining whether MHO represents a transient phenotype that is affected by aging, behavioral, and environmental factors, and accurately calculating the true health risks associated with MHO remain viable topics for diligent study through properly designed and conducted longitudinal studies.

The Bogalusa Heart Study examined 1098 individuals, both children (5–17 years) and adults (24–43 years), who participated in the study between 1997 and 2002. Participants with the MHO phenotype during childhood were more likely to maintain MHO status in adulthood. Despite the level of obesity and fat mass continuing to increase throughout childhood and adulthood, this group of MHO individuals showed a generally comparable cardiometabolic profile with non-obese children and adults. In addition, the carotid intima-media thickness did not differ in adulthood among previous MHO children and non-obese children. These results are very important because they show that the MHO phenotype that starts in childhood and continues into adulthood may have a very favourablecardiometabolic risk profile [69]. Even more importantly, there is increasing evidence showing that the metabolic profile of MHO individuals is almost indistinguishable from that of lean individuals [4,7,70].

To date it is not at all clear whether the MHO phenotype decreases morbidity and mortality associated with obesity. MHO patients have a lower prevalence of risk factors for the development of certain diseases. For example, the intima-media thickness as a risk marker of atherosclerosis is significantly different between individuals with the MHO phenotype and metabolically healthy non-obese patients [5,12,70]. However, a recent meta-analysis has evaluated eight studies comparing mortality data from any cause or cardiovascular events in six groups of patients defined by BMI category (*n* = 61,386) and metabolic comorbidities, with a 10-year follow up. Analyses revealed that MHO individuals had an increased risk (relative risk (RR): 1.24) compared with MHNW subjects, but this was only detected in studies of 10 or more years of follow-up. In addition, all metabolically unhealthy subjects, regardless of their weight, had an increased risk compared with healthy lean individuals. Curiously in metabolically unhealthy patients, the normal-weight individuals had a higher risk (RR: 3.13), followed by overweight (RR: 2.70) and finally obese subjects (RR: 2.65) [71]. Similar results were published, indicating that both MHO and MUO individuals ultimately had a higher risk of mortality [72].

This evidence, too, is conflicting, with the underlying question being whether MHO presentations simply represent an early snapshot along the timeline of metabolic health. One group found that nearly 50% of MHO patients transitioned to metabolically unhealthy phenotypes when followed longitudinally for 10 years. Therefore, at present, it is not clear whether MHO individuals will remain such with the passage of time, or if their cardiovascular morbidity and mortality is comparable to that of non-obese subjects.

## 6. Conclusions

MHO is common among the obese population and constitutes a unique subset of characteristics that reduce metabolic and cardiovascular risk factors despite the presence of excessive fat mass. The protective factors that grant a healthier profile to individuals with MHO are being elucidated.

Despite the knowledge that visceral fat deposition is the seminal factor that ultimately causes insulin resistance and the detrimental inflammatory and hormonal profile that contributes to increased risk for cardiovascular disease, it remains unknown whether MHO has genetic predisposing factors, and whether MHO ultimately succumbs to insulin resistance and the metabolic syndrome.
